# Development of a dual channel detection system for pan-genotypic simultaneous quantification of hepatitis B and delta viruses

**DOI:** 10.1080/22221751.2024.2350167

**Published:** 2024-04-30

**Authors:** Yongzhen Liu, Stephanie Maya, Sebastian Carver, Aoife K. O’Connell, Anna E. Tseng, Hans P. Gertje, Kathleen Seneca, Ronald G. Nahass, Nicholas A. Crossland, Alexander Ploss

**Affiliations:** a Department of Molecular Biology, Princeton University, Princeton, NJ, USA; b National Emerging Infectious Diseases Laboratories, Boston University, Boston, MA, USA; c Infectious Disease Care, Hillsborough, NJ, USA; d Department of Pathology and Laboratory Medicine, Boston University Chobanian & Avedisian School of Medicine, Boston, MA, USA; e Department of Virology, Immunology, & Microbiology, Boston University Chobanian & Avedisian School of Medicine, Boston, MA, USA

**Keywords:** Hepatitis B virus, hepatitis delta virus, viral hepatitis, viral detection, humanized mice

## Abstract

Hepatitis B virus (HBV) infection remains a major public health problem and, in associated co-infection with hepatitis delta virus (HDV), causes the most severe viral hepatitis and accelerated liver disease progression. As a defective satellite RNA virus, HDV can only propagate in the presence of HBV infection, which makes HBV DNA and HDV RNA the standard biomarkers for monitoring the virological response upon antiviral therapy, in co-infected patients. Although assays have been described to quantify these viral nucleic acids in circulation independently, a method for monitoring both viruses simultaneously is not available, thus hampering characterization of their complex dynamic interactions. Here, we describe the development of a dual fluorescence channel detection system for pan-genotypic, simultaneous quantification of HBV DNA and HDV RNA through a one-step quantitative PCR. The sensitivity for both HBV and HDV is about 10 copies per microliter without significant interference between these two detection targets. This assay provides reliable detection for HBV and HDV basic research in vitro and in human liver chimeric mice. Preclinical validation of this system on serum samples from patients on or off antiviral therapy also illustrates a promising application that is rapid and cost-effective in monitoring HBV and HDV viral loads simultaneously.

## Introduction

Hepatitis B virus (HBV) infection remains a major public health problem as the etiological agent for acute and chronic hepatitis B with about 2 billion people exposed to this virus and at least 257 million individuals living with chronic hepatitis B (CHB) worldwide [1]. Long-term HBV infection can lead to ongoing intrahepatic inflammation, activation of liver fibrogenic processes, and eventual progression to decompensated severe liver disease or hepatocellular carcinoma (HCC). Even though nucleos(t)ide analogs (NAs) including entecavir (ETV), tenofovir disoproxil fumarate (TDF), tenofovir alafenamide (TAF) and the less commonly utilized pegylated type I interferon (IFN) are currently available for antiviral therapies [2], HBV infection is rarely cured and may require lifelong treatment due to the persistence of HBV covalently closed circular DNA (cccDNA) – the major template for HBV gene transcription. The main goal of the current therapy is to inhibit HBV replication and improve CHB patient survival and quality of life by preventing HBV-related disease progression and decrease the risk of HCC development to reduce the morbidity and mortality of CHB. Ergo, prevailing clinical strategies aim to achieve sustained HBV suppression or even undetectable HBV DNA in CHB patients [3]. However, in addition to abdominal ultrasound examination for the risk of disease progression and HCC, HBV DNA levels need to be routinely monitored in these patients to detect possible recurrent viremia [4]. HBV functional cure, defined by HBsAg loss in CHB patients, is currently infrequently achieved and even in patients with undetectable circulation HBV DNA, HBsAg still persists at high levels attributable to the persistent cccDNA reservoir in the nucleus of hepatocytes and HBV genome integration [3–5].

Hepatitis delta virus (HDV), the sole member of the *Deltavirus* genus, is a small circular single-stranded negative sense RNA (∼1700 nucleotides) virus which can cause hepatitis D [6]. The genome of HDV is variable, G + C rich (∼60%) and has high level of base pairing with extensive secondary structures. Eight major genotypes spanning numerous global regions [7] have been characterized, between which they may differ by as much as ∼30% in nucleotide sequence. HDV is a satellite virus of HBV because it depends on HBV surface proteins (HBsAg) for its virion assembly and release, and thus HDV spread relies on co-infection with HBV [8]. HDV can be established through superinfection of HBV carriers or coinfection when both viruses infect a same individual simultaneously. Recent meta-analyses indicate that as many as 13.02–14.57% of the HBsAg-positive population, corresponding to about 50–72 million patients, are coinfected with HDV globally [9, 10]. These percentages are even higher in certain risk groups such as people who inject drugs (PWID, 37.57%) and those engaging in higher risk sexual activity (17.01%) [9].

Compared with chronic HBV infection alone, HBV and HDV dual infection is frequently associated with the most severe form of viral hepatitis, accelerated liver disease progression, and can lead to the development of cirrhosis in 70–80% of cases within 5–10 years. Moreover, upwards of 50% of patients die from liver disease within 10 years of diagnosis [7, 11]. The rates of HCC and hepatic decompensation also are about 2–3-fold higher than for HBV mono-infection [7]. Unfortunately, currently no treatments for HDV infection are approved by the US Food and Drug Administration (FDA) despite pegylated interferon α being used in most countries. Unlike the anti-HBV NAs drugs which can repress and control HBV replication at very low levels by inhibiting HBV polymerase, the NAs do not inhibit HDV replication because HDV uses host enzymes to propagate its genome [12, 13]. Fortunately, the HDV entry-inhibitor bulevirtide received conditional marketing authorization in the European Union (EU) in 2020 (Hepcludex) [14] and it is in phase 3 clinical trials in the United States.

Clinically, the presence of HDV antibodies in the blood is used to diagnose ongoing or prior HDV infection, while an active infection can only be diagnosed by detection of HDV RNA. However, due to the lack of a reliable and universal HDV RNA quantification assay, the HDV RNA determination system is not well established yet; only about 20% to 50% of HDV-infected patients have been diagnosed due to the lack of disease awareness and limited access to reliable diagnostic tests [11]. Given that HBV DNA levels in blood are tested as the gold standard biomarker to evaluate the virological response under anti-HBV treatment, with the approval of anti-HDV drugs, a standard HBV DNA and HDV RNA detection system is necessary to monitor both viral responses simultaneously to monitor the efficacy of clinical treatment protocols.

In this study, we established a probe-based, cost-effective dual channel detection system that can specifically quantify pan-genotypic HBV DNA and HDV RNA simultaneously in a single reaction. The application of this system was confirmed for HBV and HDV detection *in vitro*, in human liver chimeric mice, and was preclinically assessed utilizing patient sera.

## Materials and methods

*Cell lines.* 293 T (American Tissue Culture Collection, ATCC® Number: CRL-3216TM, Manassas, VA) and HepG2 cell lines (American Tissue Culture Collection, ATCC® Number: HB-8065™, Manassas, VA) were maintained in Dulbecco’s Modified Eagle Medium (DMEM; ThermoFisher, Waltham, MA) base medium supplemented with 10% (vol/vol) fetal bovine serum (FBS) (Sigma Aldrich, St Louis, MO). 293 T cells were grown on tissue culture-treated plastic ware (Corning Inc., Corning, NY) and HepG2 cells on type IV collagen-coated plates (Sigma Aldrich, St Louis, MO).

*HBV and HDV dual channel one-step quantitative PCR.* HBV and HDV qPCR primers were designed in genomic regions that are highly conserved among different genotypes obtained from Azenta Life Sciences (Burlington, MA). Both HBV and HDV probes were designed based on Affinity Plus^TM^ qPCR probe technology to impart heightened structural stability, leading to increased hybridization melt temperature (T_m_) and were obtained from Integrated DNA Technologies (Coralville, IA). The primer and probe sequences and their 5’ fluorophore reporter dyes, 3’ quencher are listed in Supplementary Table 1. For the One-Step quantitative PCR, the Luna® Universal Probe One-Step RT-qPCR kit was used at a final concentration of 0.4 μM primers and 0.2 μM probes in the single reaction system for HBV and HDV, respectively. The PCR was performed at 55°C for 10 mins, 95°C for 1 min followed by 45–50 cycles at 95°C for 15s, and 61°C for 40s for signal capture.

*Correlation and regression assays.* The correlation and regression analysis of the quantifications between HBV-HDV dual and the HBV or HDV single detection system were performed using GraphPad Prism (Version 10.1.1, Boston, MA) software. Simple linear regression was used and goodness of fit was characterized by the R squared values from the Pearson r. 95% confidence intervals shown as dashed lines on the graphs. Two-tailed *p* values are shown to indicate the significance (alpha = 0.05) of the correlation.

*HBV and HDV transfection of human hepatoma cells*. All work with infectious HBV and HDV has been reviewed and approved by the Princeton University Institutional Biosafety Committee (#1146). HepG2 cells were used for all transfection assays. Briefly, 1.2E + 5 or 7.0E + 4 cells/well were seeded in collagen-coated 24-well or 48-well plates, respectively. Within 24 h of seeding plates, cells were transfected with HBV-, HDV-encoding or empty backbone plasmids using Opti-MEM™ Reduced Serum Medium (Thermo Fisher Scientific, Waltham, MA) and X-tremeGENE™ HP DNA Transfection Reagents (St. Louis, MO). Total amount of transfected DNA was set to 0.5 μg/well for 24-well plates and 0.3 μg/well for 48-well plates. Six hours post transfection, the cells were washed at least 4 times. Supernatants were harvested on day 3 post transfection.

*HDV full-length RNA in vitro transcription.* HDV full-length DNA template was PCR amplified through the pSVL(D3) [15] plasmid (kindly provided by Dr. John Taylor, Fox Chase Cancer Center) by HDV-T7-F (TAATACGACTCACTATAGGCTTGGGCTCGGGCGGCG) and HDV-R (CTTGAGCCAAGTTCCGAGCGAG). Then, the full-length 1.0x HDV DNA was purified and inserted into a TOPO-Blunt Cloning vector (Thermo Fisher, Waltham, MA) and the correct clone was then picked and plasmid DNA prepared using a miniprep kit (MACHEREY-NAGEL, Allentown, PA). The in vitro transcription template was then PCR amplified using the constructed vector with the same primers above and the full-length 1.0x HDV RNA was *in vitro* transcripted using a HiScribe T7 High Yield RNA Synthesis Kit (New England Biolabs, Ipswich, MA) following manufacturer’s instructions. To remove template DNA, 70 μl nuclease-free water was added to the reaction system and then digested with DNase I (New England Biolabs, Ipswich, MA) for 15 min at 37 °C. The RNA was then cleaned up through the MEGAclear kit (Thermo Fisher Scientific, Waltham, MA) following manufacturer’s instructions. The purified HDV RNA was confirmed by running an RNA gel (Supplementary Fig. 1A) and the concentration was measured using a NanoDrop spectrophotometer (Thermo Fisher Scientific, Waltham, MA) and the copy number was calculated based on its molecular weight.

*HBV minicircle standard preparation.* The HBVcircle was prepared by using the parental construct in combination with the minicircle *E. coli* strain which has been specifically engineered to express the ϕ31 integrase and Scel endonuclease upon arabinose induction [16]. Briefly, the HBV minicircle parental plasmid transformed colony was precultured for about 6 h in LB medium containing 50 μg/mL Kanamycin and then was transferred and cultured overnight in 400 mL TB medium with 50 μg/mL Kanamycin. When the OD600 of the overnight culture is between 4 and 5, a minicircle induction mix composed of 400 mL LB broth, 16 mL 1N NaOH and 400 μL 20% L-arabinose was combined with the bacteria medium and was incubated at 30 °C with shaking at 250 rpm for another 5 h or longer (Supplementary Fig. 1A). Bacteria were pelleted and the HBVcircle DNA was extracted and purified using a NucleoBond Xtra Midi kit (MACHEREY-NAGEL, Allentown, PA). To confirm the HBVcircle, the DNA with or without EcoR I digestion was run on a gel to see the supercoiled HBVcircle and the linearized 1.0x HBV genome (Supplementary Fig. 1B&C). The HBVcircle was further characterized by transfecting into HepG2 cells to see the HBsAg production and HBV DNA levels in the supernatants (Supplementary Fig. 1D).

*Phylogenetic analysis of HBV and HDV genome variety among genotypes.* The HBV DNA and HDV RNA sequences of different genotypes were retrieved from the National Center for Biotechnology Information (NCBI) and three representative genomes were used for each genotype except for HDV genotypes 1 and 6 for which an insufficient number of genome sequences were available. The phylogenic trees were built by MegAlign Pro (DNASTAR Inc., Madison, WI) using a RAXML alignment of HBV DNA or HDV RNA sequences among genotypes, respectively. The nucleotide numbering is based on the genome in the first line and different nucleotides are coloured. The matrix of genome identity and genetic distance were shown in the Supplementary Fig. 3.

*Quantification of HBsAg and HBeAg*. Detection and quantification of HBsAg and HBeAg levels were performed by a chemiluminescence immunoassay (CLIA) according to the manufacturer’s instructions (Autobio Diagnostics CO., LTD, Zhengzhou, Henan, China) and the chemiluminescence was determined by a TriStar Multimode Reader (Berthold Technologies, Bad Wildbad, Germany).

*DNA and RNA isolation from cell supernatants and mouse sera.* The DNA and RNA were isolated using the QIAamp MinElute Virus Spin Kit (Qiagen, Hilden, Germany), following the manufacturer’s instructions. Briefly, 20 μL were processed per sample and the purified DNA and RNA was eluted in 20 μL. To prepare supernatants for qPCR after infectious clone transfection, possible plasmid contamination was removed by DNase I (Thermo Fisher Scientific, Waltham, MA) treatment (37°C for 1 h) prior to purification. Samples were then stored at −80°C until further use.

*Western blotting*. Cells which had been seeded in a 24-well plate were lysed with 150 μL/well of 1X NuPAGE LDS buffer (Life Technologies, Carlsbad, CA) containing 2.5% (vol/vol) 2-mercaptoethanol (β-ME) at room temperature for 10 mins. The cell lysate was then transferred to a sterile 1.5 ml tube and heat-inactivated at 95°C for 20 min. The samples were then loaded and separated by 10% (w/vol) sodium dodecyl sulfate-polyacrylamide gel electrophoresis (SDS-PAGE), and transferred to a nitrocellulose membrane (Roche, Basel, Switzerland). The membrane was blocked with 5% (w/vol) skim milk in Tris-buffered saline containing 0.1% Tween-20 (TBST) for 1 h at room temperature and then incubated overnight at 4°C with the HBV core protein primary antibody (1:1000 dilution, rabbit polyclonal antibody, kindly provided by Dr. Ju-Tao Guo, Blumberg Institute, Doylestown, PA) or HDAg antibody (1:1000 dilution, rabbit polyclonal antibody, a gift from John Taylor, Fox Chase Cancer Center, Philadelphia, PA). Next, the membranes were washed three times with 1X (Tris-Buffered Saline, 0.1% Tween® 20 Detergent) TBST and incubated with a goat anti-Rabbit IgG (1:5000 dilution; Thermo Fisher Scientific, Waltham, MA) secondary antibody at room temperature for 1 h. After further washing with TBST, the signal intensities of the membranes were scanned and visualized using an Odyssey CLx imager (Li-COR Biosciences, Lincoln, NE). The signal quantification was performed by ImageJ.

*Generation of cell-culture derived HBV and HDV stocks by transfection*. For HBV stocks, HepG2 cells were seeded into two 150-mm collagen-coated plates at 7.0E + 6 per plate. When cell confluence reached approximately 90%, the medium was replaced with 25 mL DMEM/F12 (Life Technologies, Carlsbad, CA), supplemented with 5% (vol/vol) FBS, 1% (vol/vol) penicillin/streptomycin (P/S) prior to DNA transfection. Cells in each plate were transfected with 28 μg plasmid DNA mixed with 2800 μL Opti-MEM™ Reduced Serum Medium (#51985034, Thermo Fisher Scientific, Waltham, MA) and 84 μL X-tremeGENE™ HP DNA Transfection Reagent (St. Louis, MO). After 4–6 h, cells were washed three times with 25–30 mL pre-warmed Dulbecco’s Phosphate Buffered Saline (DPBS) and then cultured in 20 mL DMEM/F12 supplemented with 5% (vol/vol) FBS, 1% (vol/vol) P/S. After 2 h, cells were again washed twice with pre-warmed 25–30 mL DPBS. Cells were subsequently cultured in 20 mL DMEM/F12 supplemented with 5% (vol/vol) FBS, 1% (w/vol) P/S. Supernatants were harvested every 2–3 days for 10–11 days and stored at 4°C. Supernatants were centrifuged at 3000*g* for 15 min at 4°C to remove cell debris. Thereafter, supernatants were transferred into clean tubes and concentrated using heparin columns (GE Healthcare, Chicago, IL). Briefly, supernatants were run through the 5 mL heparin columns at a speed of 5 mL/min. Then the columns were washed once with 8 mL 1×PBS followed by elution with 30 mL elution buffer (25 mL 10×PBS pH 7.4 diluted into 100 mL with 75 mL distilled water). The elution was collected and further concentrated with the centrifugal filters (100,000 NMWL, Merck Millipore Ltd.) at 3000 *g* for 30 min at 4°C. Finally, about 1.6 mL virus stocks were transferred and aliquoted in LoBind Microcentrifuge Tubes (Eppendorf, Hamburg, Germany). Then 20 μL of virus stock was used and DNase I (Thermo Fisher Scientific, Waltham, MA) was added (37°C, 1 h) to digest possible residual plasmid DNA. DNA was extracted with the QIAmp MinElute virus spin kit (Qiagen, Hilden, Germany). Thereafter, HBV titres were quantified by qPCR as described here. Based on the HBV DNA copy numbers virus was aliquoted and cryopreserved at −80 °C until use. For HDV stocks, the production process was the same with HBV exception that 14 μg of each the HDV pSVL(D3) and HBV preS1 overexpression plasmids were transfected.

*Generation of HepG2-hNTCP infection cell model.* Lentiviral hNTCP pseudo-particles were generated as previously described [17]. Briefly, 4.0E + 6 293 T cells were seeded in a 10 cm tissue-culture plate and plasmids expressing the respective pLVX-hNTCP-tagRFP proviral DNA, HIV-1 gag–pol, and VSV-G at a ratio of 1/0.8/0.2 were co-transfected using Xtremegene (Sigma-Aldrich, St. Louis, MO). Supernatants were collected at 24, 48 and 72 hpurs post transfection, pooled, and filtered through a 0.45 μm filter (Millipore, Burlington, MA). Filtered lentiviral supernatants were supplemented with polybrene (MilliporeSigma, Darmstadt, Germany) (4 μg/mL, vol/vol) and (1:50, vol/vol) 1 M HEPES (Life Technologies, Carlsbad, CA), aliquoted, and stored at −80 °C until use. Next, HepG2 cells were transduced with the hNTCP-tagRFP encoding lentiviruses. After 3 days, expression of the fusion protein was assessed by both confocal microscope (Nikon A1R-STED, Nikon, Melville, NY) in the Princeton University Imaging Core facility and LSRII Multi-Laser Analyzer (BD, Franklin Lakes, NJ) at the Princeton University flow cytometry core facility. The flow cytometry data was analysed by FlowJo (Version 10.4, BD Biosciences, Franklin Lakes, NJ).

*HBV and HDV infection of HepG2-NTCP cells*. HepG2-NTCP cells were seeded into 48-well plates at 5.0E + 4 cells/well, and then were pre-treated with pretreat medium (DMEM supplemented with 3% (vol/vol) FBS, 2% (vol/vol) DMSO, 1% (vol/vol) Pen/Strep) for 24 h. HBV infections at a multiplicity of infection (MOI) of 8000 and HDV infections at a MOI of 1000 were proceeded in the presence of 4% polyethylene glycol (PEG) 8000 (Sigma-Aldrich, St. Louis, MO) and 2% dimethyl sulfoxide (DMSO, Sigma-Aldrich, St. Louis, MO). 12–24 h following inoculation, the cells were washed with pre-warmed sterile 1 × PBS at least 4 times and then incubated with fresh maintenance media (DMEM supplemented with 3% FBS, 2% DMSO, 1% Pen/Strep and 1x non-essential amino acids). Samples were collected at the indicated time-points and stored at −80°C until analysis.

*In vivo experiments.* All animal experiments were performed in accordance with protocols reviewed and approved by the Institution Animal Care and Use Committee (IACUC) of Princeton University (protocol number 3063).

The generation of Fah-/- NOD.Cg-Rag1^tm1MomIL2rgtmlWjl/SzJ^ IL2Rg^null^ (FNRG) mice has been previously described [18, 19]. Female FNRG mice older than 6 weeks of age were transplanted with ca. 1.0E + 6 cryopreserved adult human hepatocytes. Primary hepatocytes were obtained from BioReclamation Inc. (Westbury, NY). FNRG mice were cycled on water supplemented with 2-(2- nitro-4-trifluoromethylbenzoyl)-1,3-cyclohexanedione (NTBC, Yecuris Inc., Tualatin, OR), to block the build-up metabolites to toxic concentrations. Approximately 3 months after transplantation, blood was collected by submandibular puncture and serum was separated by centrifuging the coagulated blood at 1100 × g for 15 min. Mouse sera were analysed via human albumin ELISA, and mice with a serum concentration of ≥1000 μg/mL human albumin were chosen for the subsequent viral infections. For HBV mono-and for HBV-HDV super-infection, 3.0E + 8 GE/animal HBV (200 μl volume with sterile 1X PBS) was inoculated by intraperitoneal (i.p) injection. In the HBV-HDV super-infection group, human chimeric mice were inoculated with 5.0E + 8 GE/mouse HDV by i.p infection (200 μl volume diluted in sterile 1X PBS).

*Human albumin ELISA for assessment of human hepatocyte engraftment of chimeric mice.* Levels of human albumin in mouse serum were quantified by ELISA as described previously [20]. Briefly, 96-well flat-bottomed plates (Nunc, Thermo Fisher Scientific, Waltham, MA) were coated with goat anti-human albumin antibody (1:500, #A80-129A, Thermo Fisher Scientific, Waltham, MA) in coating buffer (1.59 g Na_2_CO_3_, 2.93 g NaHCO_3_, 1L dH2O, pH = 9.6) for 1 h at 37°C. The plates were washed four times with wash buffer (0.05% (vol/vol) Tween 20 (Sigma Aldrich, St. Louis, MO) in 1X PBS), and then incubated with superblock buffer (Fisher Scientific, Hampton, NH) for 1 h at 37°C. Plates were washed twice. Human serum albumin (Sigma Aldrich, St. Louis, MO) was diluted to 1 μg/mL in sample diluent (10% Superblock, 90% wash buffer), then serially diluted 1:2 in 135 μL sample diluent to establish an albumin standard. Mouse serum (5 μl) was used for a 1:10 serial dilution in 135 μL sample diluent. The coated plates were incubated for 1 h at 37°C, then washed three times. Mouse anti-human albumin (50 μL, 1:2000 in sample diluent, Abcam, Cambridge, UK) was added and plates were incubated for 2 h at 37°C. Plates were washed four times and 50 μL of goat anti-mouse-horse radish peroxidase (HRP, 1:10,000 in sample diluent, LifeTechnologies, Carlsbad, CA) were added and incubated for 1 h at 37°C. Plates were washed six times. Tetramethylbenzidine (TMB, 100 μL) substrate (Sigma Aldrich, St. Louis, MO) was added and the reaction was stopped with 25 μL 2-N H_2_SO_4_. Absorbance was read at 450λ on the BertholdTech TriStar (Bad Wildbad, Germany).

*Quantification of HBV pgRNA by quantitative RT–PCR*. HBV RNA purified from supernatants was used to determine extracellular nucleocapsid-associated HBV pre-genome RNA (pgRNA) level as described previously [21]. Briefly, 7.5 μl of isolated nucleic acid was treated with DNase I (Thermo Fisher Scientific, Waltham, MA) followed by reverse transcription with a specific HBV primer (5’-CGAGATTGAGATCTTCTGCGAC-3’, nt 2415-2436, numbered based on gt D with GenBank accession no. U95551.1) located in precore/core region [21] and anchored sequences using RevertAid^TM^ First Strand DNA Synthesis Kit (Thermo Fisher Scientific, Waltham, MA, USA). For quantitative assays, the standards with 1-mer HBV target template were cloned into the TOPO-Blunt Cloning vector (Thermo Fisher, Waltham, MA, USA #450245) and the copy number was calculated based on the number of nucleotides of the plasmid and concentration of the plasmid preparation. A master mix was created containing 15 µl 2× Taqman reaction mix (Applied Biosystems, Waltham, MA, USA), 500 nM forward and reverse primers, 200 nM probe and 3 µl synthesized cDNA in a 30 µL reaction. The master mix was then added to the samples or to the 10-fold serial dilution standards and the following cycling programme was used to run the qPCR: 95°C for 10 min; 45 cycles at 95°C for 15 sec and 58°C for 45 sec.

*Immunofluorescence of HBcAg and HDAg.* For infection samples, HepG2 cells +/- NTCP were grown on collagen-precoated cover slides and fixed 6 days after infection with 4% (vol/vol) paraformaldehyde (PFA). For transfection samples, HepG2 cells were grown on collagen-precoated cover slides and fixed 3 days after transfection with 4% (vol/vol) PFA. The cells were then washed 3 times with 1 × PBS followed by 1 h of incubation with blocking and permeabilization buffer (5% BSA, 5% FBS, 0.3% Triton-X-100 in 1 × PBS, all (vol/vol)) at room temperature. Thereafter, cells were incubated with either anti-HBc antibody (1:500 dilution, #B0586, Dako, Denmark) or anti-HDAg (1:250 dilution, a gift from John Taylor, Fox Chase Cancer Center, Philadelphia, PA), respectively in the dilution buffer (1% BSA, 1% FBS, 0.3% Triton-X-100 all (vol/vol) in 1 × PBS) at 4°C overnight, washed for 3 times and incubated with an Alexa Fluor^®^ 488 conjugated goat anti-rabbit secondary antibody (1:1000 dilution, #A11008, Thermo Fisher Scientific, Waltham, MA) and DAPI (1 µg/mL) in dilution buffer (1% BSA, 1% FBS, 0.3% Triton-X-100 all (vol/vol) in 1 × PBS) at room temperature for 1 h. After 3 washes, the cover slides with cells were then transferred onto a glass slide with 2 µl ProLong Gold antifade solution (#P36930, Thermo Fisher Scientific, Waltham, MA) and sealed with CoverGrip™ Coverslip Sealant (#23005, Biotium Inc., Fremont, CA). The slides were then covered with foil and dried overnight at 4°C and subsequently analysed with a confocal microscope (Nikon A1R-STED, Nikon, Melville, NY) in the Imaging Core facility at Princeton University.

*Histology processing and Multiplexed HDAg, HBcAg and human β2-macroglobulin immunohistochemistry.* Tissue samples were fixed for a minimum of 72 h in 4% (w/vol) paraformaldehyde (PFA) before processing in a Tissue-Tek VIP-5 automated vacuum infiltration processor (Sakura Finetek USA, Torrance, CA) and embedded in paraffin using a HistoCore Arcadia paraffin embedding machine (Leica, Wetzlar, Germany). 5-μm tissue sections were generated using a RM2255 rotary microtome (Leica) and transferred to positively charged slides, deparaffinized in xylene, and dehydrated in graded ethanol. A Ventana Discovery Ultra tissue autostainer (Roche, Basel, Switzerland) was used for multiplex fluorescent immunohistochemistry (mfIHC). Briefly, tyramide signal amplification (TSA) was used in an iterative approach to covalently bind Opal fluorophores (Akoya Bioscience, Marlborough, MA) to tyrosine residues in tissue. Sequential heat-stripping was performed on each primary-secondary antibody complex following each fluorophore application until all antibodies were developed. Liver tissue from non-humanized and non-infected FNRG mice was used as a negative control for the immunohistochemistry assay to confirm the specificity of antibodies. Details for the three-plex fmIHC assays developed for this work are outlined in Supplementary Table 2. All primary antibodies were of rabbit origin, and thus developed with a secondary goat anti-rabbit HRP-polymer antibody (Vector Laboratories, Burlingame, CA) for 20 min at 37°C. All Opal TSA-conjugated fluorophore reactions took place for 20 min. Slides were counterstained with spectral DAPI (Akoya Biosciences, Marlborough, MA) for 16 min before being mounted with ProLong Gold Antifade Mountant (ThermoFisher, Waltham, MA).

*Multispectral microscopy.* Fluorescently labelled slides were imaged using a Vectra Polaris^TM^ Quantitative Pathology Imaging System (Akoya Biosciences, Marlborough, MA). Exposures for all Opal dyes were established utilizing regions of interest with strong signal intensities to minimize exposure times and maximize the specificity of the signal detected. To maximize signal-to-noise ratios, images were spectrally unmixed using a synthetic library specific for each Opal fluorophore and DAPI. Furthermore, an unstained section was used to create an autofluorescence signature that was subsequently removed from whole-slide images using InForm software version 2.4.8 (Akoya Biosciences, Marlborough, MA). InForm exported the multispectrally unmixed images as QPtiffs, which were then fused together as a single whole slide image in HALO (Indica Labs, Albuquerque, NM) for acquisition of representative images.

*Serum samples from HBV and HBV/HDV positive patients.* Serum samples from deidentified HBV + patients were obtained from the American Red Cross, and samples from HBV+/HDV + patients from ID Care. This work was deemed non-human subjects research by the Princeton University Institutional Review Board (IRB).

*HBV genome cloning from patient serum.* HBV DNA was isolated by processing 20 μL patient serum through the QIAamp MinElute Virus Spin Kit (Qiagen, Hilden, Germany). HBV full-length genomes were then PCR-amplified using Hi-fidelity polymerase (PrimeSTAR® GXL DNA Polymerase, Takara Bio USA, San Jose, CA) by HBV-F (5’-CCGGAAAGCTTGAGCTCTTCTTTTTCACCTCTGCCTAATCA-3’) and HBV-R (5’-CCGGAAAGCTTGAGCTCTTCAAAAAGTTGCATGGTGCTGG-3’) which can anneal to regions that are conserved across the different HBV genotypes. The PCR product was then purified and then inserted into a TOPO-Blunt Cloning vector (Thermo Fisher, Waltham, MA) following by Sanger sequencing (Azenta Life Sciences, Burlington, MA).

*Statistical Analysis.* Bar graphs were presented as mean ± SEM with individual data points and at least three biological independent replicates were performed. All data was analysed with Prism v9.2.0 (GraphPad) and Student’s t test (unpaired, two-tailed) was used for the statistics. Statistical significance of any *p* values is indicated.

## Results

**Figure 1.**
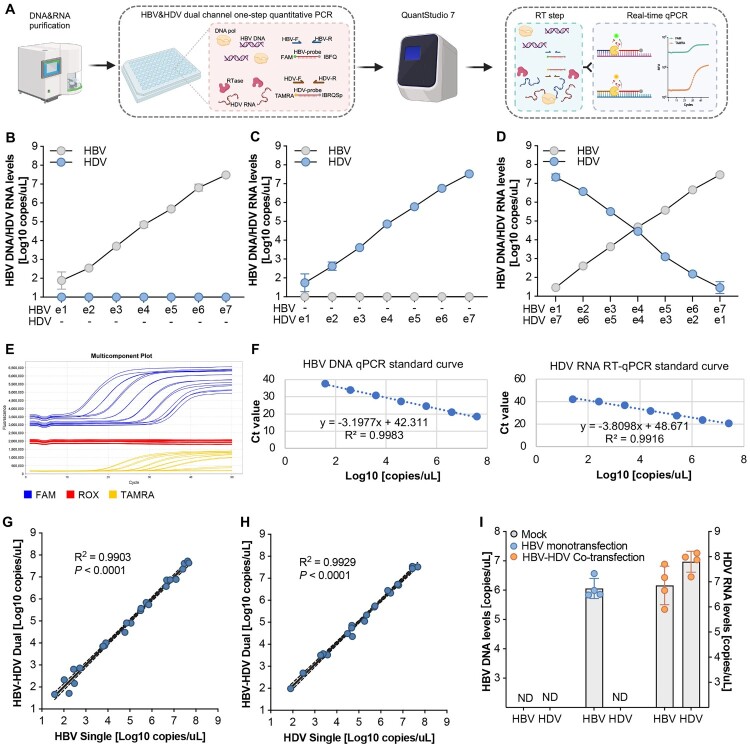
Characterization and validation of the sensitivity, specificity, and accuracy of the HBV-HDV dual channel quantitative detection system. (A) Schematic of the HBV-HDV dual channel one-step quantitative PCR. The DNA and RNA are purified from the samples and HBV DNA and HDV RNA are detected and quantified in a single reaction simultaneously by using HBV and HDV specific primers and probes, respectively. (B) Quantification of serially diluted HBV genomes by (RT-)qPCR in the absence of HDV RNA. (C) Quantification of serially diluted HDV genomes by (RT-)qPCR in the absence of HBV DNA. (D) Serial dilutions of HBV DNA and HDV RNA in the detection samples show the HBV-HDV dual channel quantitative detection system can specifically and simultaneously detect HBV DNA and HDV RNA in a single qPCR. (E) A representative multicomponent plot to show the fluorescence signal amplification in the dual detection system by using the QuantStudio 7 Flex qPCR machine. The FAM channel was used for HBV DNA, the TAMRA channel was used for HDV RNA and the Rox channel was used as the passive control. (F) Standard curves for HBV DNA (left) and HDV RNA (right) quantification. The accuracy of the HBV-HDV dual channel quantitative detection system was validated by analysing the correlations of the quantifications between HBV-HDV dual detection system and the HBV (G) or HDV (H) single detection system through simple linear regression. The *R*-squared values show the degree of their linear correlation and the *P* values show the statistical significance. The dashed lines indicate 95% confidence intervals. (I) Characterization of the HBV-HDV dual channel quantitative detection system in the transfection system with mock, HBV mono-transfection or HBV-HDV co-transfection. Data shown as mean ± SD of 4 biological replicates.

*Characterization and validation of the sensitivity, specificity, and accuracy of the HBV-HDV dual channel quantitative detection system*. The HBV-HDV dual channel quantitative detection system was developed by employing a one-step RT-qPCR system with Warm Start-activated reverse transcriptase, Hot Start DNA-dependent DNA polymerase, dNTPs, and all the necessary buffer components into a single reaction. For simultaneous quantification of HBV and HDV, specific FAM (Carboxyfluorescein) and TAMRA (Carboxytetramethylrhodamine) labelled probes were designed targeting the respective genomes (Figure 1(A)). HDV quantification standards were prepared through *in vitro* transcription of full-length negative-strand HDV RNA to closely mimic the authentic HDV genome (Supplementary Fig. 1A). Standards for HBV DNA were created using the HBV minicircle technology [22], generating 1x HBV genome molecules (Supplementary Fig. 1B, 1C, 1D). The amplification efficiency of the HBV DNA qPCR and HDV RNA RT-qPCR systems was individually confirmed (Supplementary Fig. 2A, 2B).

To assess the dual application of two fluorophores in a single reaction system, a serial dilution of HBV (ranging from 10–1e7 copies) was used as the template, without input HDV. Our results demonstrate that the HBV-HDV dual channel quantitative detection system exhibits robust target specificity, showing no carryover or contaminated fluorescence signal from the FAM channel to the TAMRA channel, even at the highest HBV levels (Figure 1(B)). This observation was validated running the assay on serially diluted HDV, demonstrating no carryover or contaminated fluorescence signal from the TAMRA channel to the FAM channel, even at the highest HDV levels (Figure 1(C)).

Further experimentation, involving gradually increased HBV templates and simultaneously decreased HDV templates, demonstrated that the HBV-HDV dual channel quantitative detection system maintained excellent readout specificity (Figure 1(D,E)) and sensitivity, displaying exceptional linear amplification across a 7-log range (1e1 to 1e7) of input template concentrations (Figure 1(F)). Correlations of quantifications between the HBV-HDV dual detection system and the HBV (Figure 1(G)) or HDV (Figure 1(H)) single detection systems indicated similar accuracy.

To validate its applicability, the HBV-HDV dual detection system was tested in a transfection culture model, demonstrating its ability to detect HBV DNA only in the supernatant of the HBV mono-transfection group. Moreover, our platform could capture and quantify both HBV DNA and HDV RNA simultaneously in the HBV-HDV co-transfection group (Figure 1(I)).

*The HBV and HDV dual channel system can be used for pan-genotype specific HBV DNA and HDV RNA detection.* HBV exhibits remarkable genetic heterogeneity owing to its error-prone reverse transcriptase/polymerase, resulting in its phylogenetic classification into at least nine genotypes (A to I) [19]. Similarly, the genetic diversity of HDV is substantial, leading to the identification of eight major clades based on phylogenetic classification derived from their genome sequences [23]. Although distinct genotypes of HBV or HDV may traditionally align with specific ethno-geographic regions, contemporary population migration and increased mobility have contributed to a more pan-genotypic prevalence globally. Consequently, the development of a universal, comprehensive pan-genotypic detection system would have great utility for monitoring HBV and HDV infections and quantifying viral loads across diverse patient populations.

**Figure 2.**
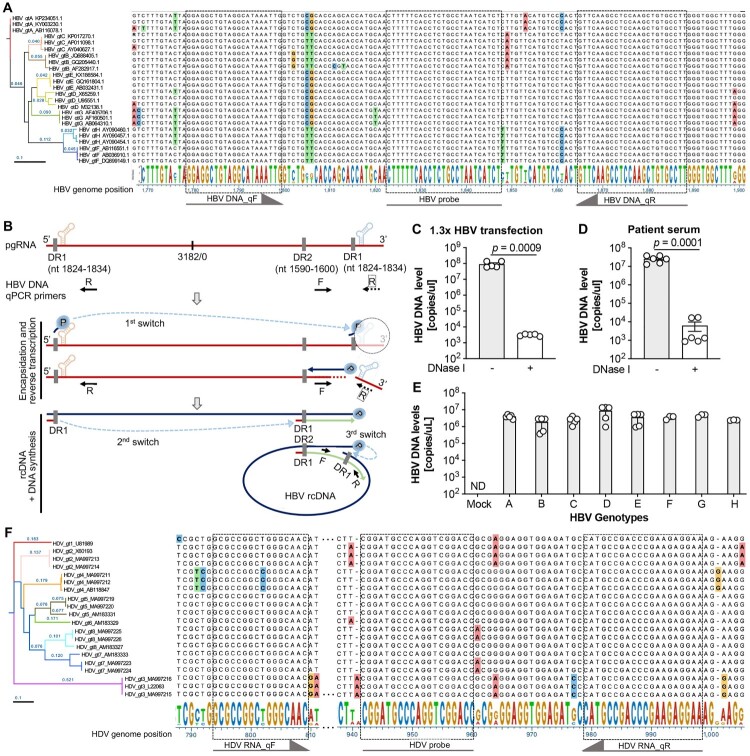
The HBV and HDV dual channel system can be used for pan-genotype HBV DNA and HDV RNA detection. (**A**) HBV quantitative PCR primer and probe designment based on the alignment and phylogenetic assay of HBV genomes of different genotypes (A–H). The phylogenetic tree (performed by DNASTAR, the phylogeny was computed using Maximum Likelihood RAxML, order ascending by distance) shows the genome difference among genotypes and for each HBV genotype three representative genomes were used. Sequence alignment was performed under the Clustal W algorithm and non-conserved nucleotides were labelled with different colours. The conserved regions were used for primers and probe designment which were indicated by dashed lines and arrows. (**B**) Schematic to show the specificity of secreted HBV rcDNA but not HBV pgRNA detection of the HBV and HDV dual channel system. HBV pgRNA was shown with the epsilon structures and the direct repeat elements (DR) in 5’ and 3’ primes. The qPCR primers for HBV DNA in the HBV and HDV dual channel system are shown accordingly based on the HBV genome position. Characterization of the HBV DNA but not HBV pgRNA specific detection by using the supernatants after HBV infectious clone transfection (**C**) and sera from HBV infected patients (**D**). Data shown as mean ± SD of 5 biological replicates (**C**) or 6 patient samples (**D**). Statistical significance was determined applying an unpaired two-tailed *t* test. (**E**) The pan-genotypic detection of HBV DNA in the HBV and HDV dual channel quantification system is validated by detecting the supernatants after HBV different genotype (A–H) plasmid transfection, respectively. Data shown as mean ± SD of at least 3 biological replicates. (**F**) HDV quantitative PCR primer and probe design based on the alignment and phylogenetic assay of HDV genomes of different genotypes (1–8). The phylogenetic tree (performed by DNASTAR, the phylogeny was computed using Maximum Likelihood RAxML, order ascending by distance) shows the sequence difference across genotypes and for each HDV genotype three representative genomes were used. Sequence alignment was performed under the Clustal W algorithm and non-conserved nucleotides were labelled with different colours. The conserved regions were used for primers and probe designment which were indicated by dashed lines and arrows.

Through phylogenetic analysis and sequence alignment of representative HBV genome sequences spanning genotypes A to H, genetic proximity was observed between genotypes A-E, while genotypes F-H exhibited greater genetic distance (Figure 2(A); Supplementary Fig. 3A). Given that HBV pre-genomic RNA (pgRNA) and partially reverse-transcribed HBV single negative-strand DNA may be encapsidated and released into the circulation in HBV patients [2, 21], we designed primers and probes for HBV quantification to specifically detect the authentic HBV relaxed circular DNA (rcDNA) genome in highly conserved regions (nt 1779–1883) across all genotypes (Figure 2(A)). After the initiation of encapsidation and reverse transcription, the polymerase (TP domain) conjugated primer translocates from the 5’ prime DR1 to the 3’ prime DR1 to start the reverse transcription (1st switch) and at the same time the corresponding pgRNA template is degraded by the RNase H domain of the polymerase, which abrogates PCR amplification for pgRNA in the one-step quantification. After reverse transcription of the HBV minus strand, the residual RNA primer has a second template switch from 5’ DR1 to the DR2 to initiate the plus strand synthesis of HBV rcDNA, on which the primers can be annealed and the PCR can be performed (Figure 2(B)). Based on the locations of the HBV primers, the reverse primer may have two binding sites on the full-length pgRNA (Figure 2(B)). The 3’ terminus of encapsidated and secreted pgRNA is truncated [24, 25], thus eliminating one of the binding sites. The orientation of paired primers, outward to each other, avoids PCR amplification from pgRNA-derived cDNA templates, thereby exclusively amplifying and detecting HBV rcDNA.

This was corroborated by using supernatants from cells transfected with a plasmid encoding a 1.3x HBV gt D genome and patient serum, where DNase I treatment resulted in a significant reduction of detected HBV DNA levels (Figure 2(C,D)). The pan-genotype specificity of HBV DNA quantification was further validated using supernatants transfected with infectious clones from genotypes A to H, respectively (Figure 2(E)).

Similarly, the HDV genome exhibits substantial variation among different genotypes, with genetically close genotypes 2 and 5 displaying only 83.79% genome identity (Figure 2(F); Supplementary Fig. 3B). Genotype 3 of HDV stands out with only approximately 70% identity compared to the remaining seven genotypes (Supplementary Fig. 3B). Primers and probes for detecting HDV RNA by RT-qPCR were carefully designed in conserved regions (nt 794–998) (Figure 2(F)). Notably, despite a single T/C nucleotide polymorphism within the forward primer for genotype 5, previous studies have demonstrated the functionality of this primer for genotype 5 HDV quantification [26]. Therefore, our HDV quantification system covers all known genotypes.

**Figure 3.**
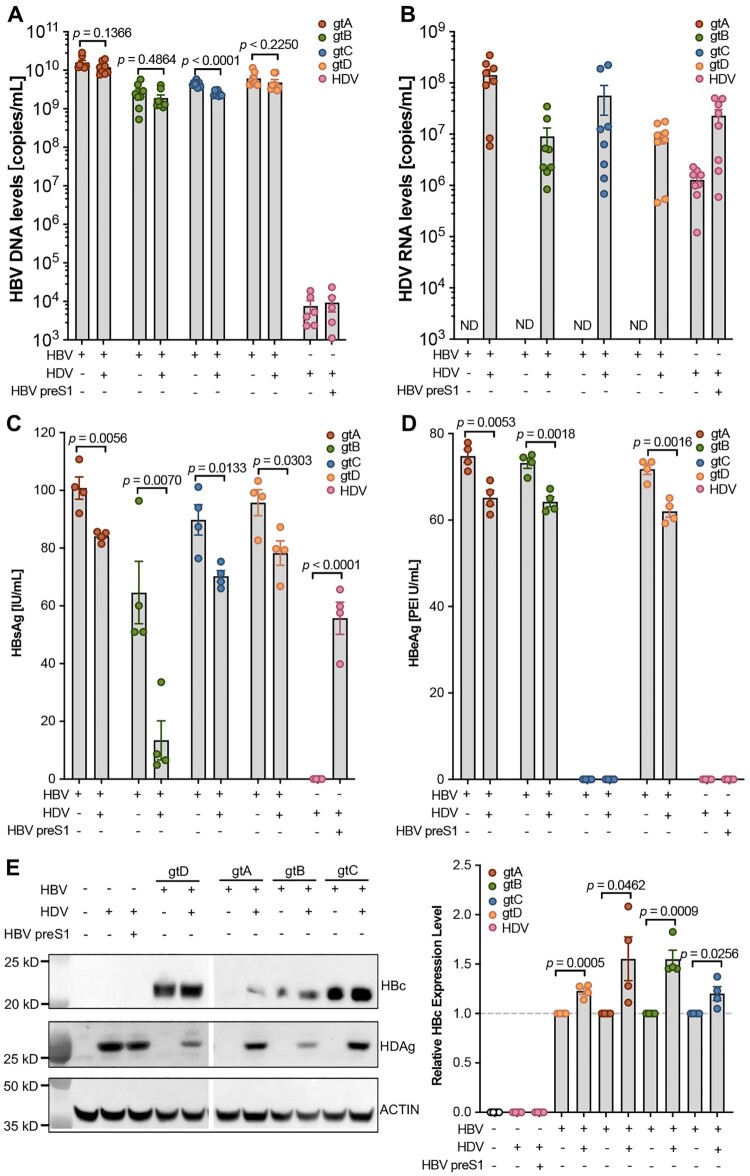
The HBV and HDV dual channel quantification system can be applied to investigate the interactions between HBV and HDV. (**A**) HBV DNA detection in the supernatants after HBV single transfection, HBV-HDV co-transfection and HDV-HBV preS1 co-transfection. (**B**) HDV RNA detection in the supernatants after HBV single transfection, HBV-HDV co-transfection and HDV-HBV preS1 co-transfection. HBsAg (**C**) and HBeAg (**D**) detection in the supernatants after HBV single transfection, HBV-HDV co-transfection and HDV-HBV preS1 co-transfection by ELISA. Statistical significance was determined applying an unpaired two-tailed *t*-test. *n* = 4. (**E**) Intracellular HBV core protein and HDV antigen were analysed by western blot after HBV single transfection, HBV-HDV co-transfection and HDV-HBV preS1 co-transfection. Data shown as mean ± SD of 2 independent western blot experiment and the quantification was performed 2 times. Statistical significance was determined applying an unpaired two-tailed *t*-test.

*The HBV and HDV dual channel quantification system can be applied to investigate the HBV and HDV interactions.* Patients with chronic hepatitis delta and B experience accelerated liver disease progression compared to those with HBV infection alone. Investigating the coexistence of HBV and HDV and their interaction within hepatocytes is crucial for understanding the underlying mechanisms of viral antigen expression, viral persistence, and gaining insights into the severe pathogenesis of HBV and HDV coinfection. Utilizing the infectious clones of HBV genotypes A to D developed previously [19] and an HDV replication-competent plasmid pSVL(D3) [15], we conducted both HBV mono-transfection and HBV and HDV co-transfection experiments on HepG2 cells. To study HDV alone, an HBV envelope protein expression vector was constructed to assemble and secrete HDV virions (Supplementary Fig. 4A). The results demonstrate the specificity of our dual-channel quantification system in detecting HBV DNA and HDV RNA, respectively (Figure 3(A,B)).

Compared with HBV mono-transfection, the levels of HBV DNA and HBV pre-genomic RNA (pgRNA) were lower when HDV was co-transfected, particularly notable for HBV genotype C (Figure 3(A); Supplementary Fig. 4B) presumably because its replication-enhancing mutations [19]. Moreover, the secretion of HBsAg and HBeAg was significantly reduced in the HBV and HDV co-transfected group (Figure 3(C,D)), suggesting that HDV may inhibit HBV replication machinery or the secretion processes. Notably, no HBeAg was detected for HBV genotype C due to basic core promoter mutations in the clinically isolated genome strain [19]. Western blot experiments demonstrated that, contrary to the decreased secretion of HBV viral markers, there was an accumulation of HBV core protein for all HBV genotypes in the HBV and HDV co-transfected groups (Figure 3(E)). Intracellular hepatitis delta antigen (HDAg) levels, on the other hand, were decreased in the presence of HBV envelope protein or HBV infectious clones, which are essential for HDV assembly and secretion (Figure 3(E); Supplementary Fig. 4C).

**Figure 4.**
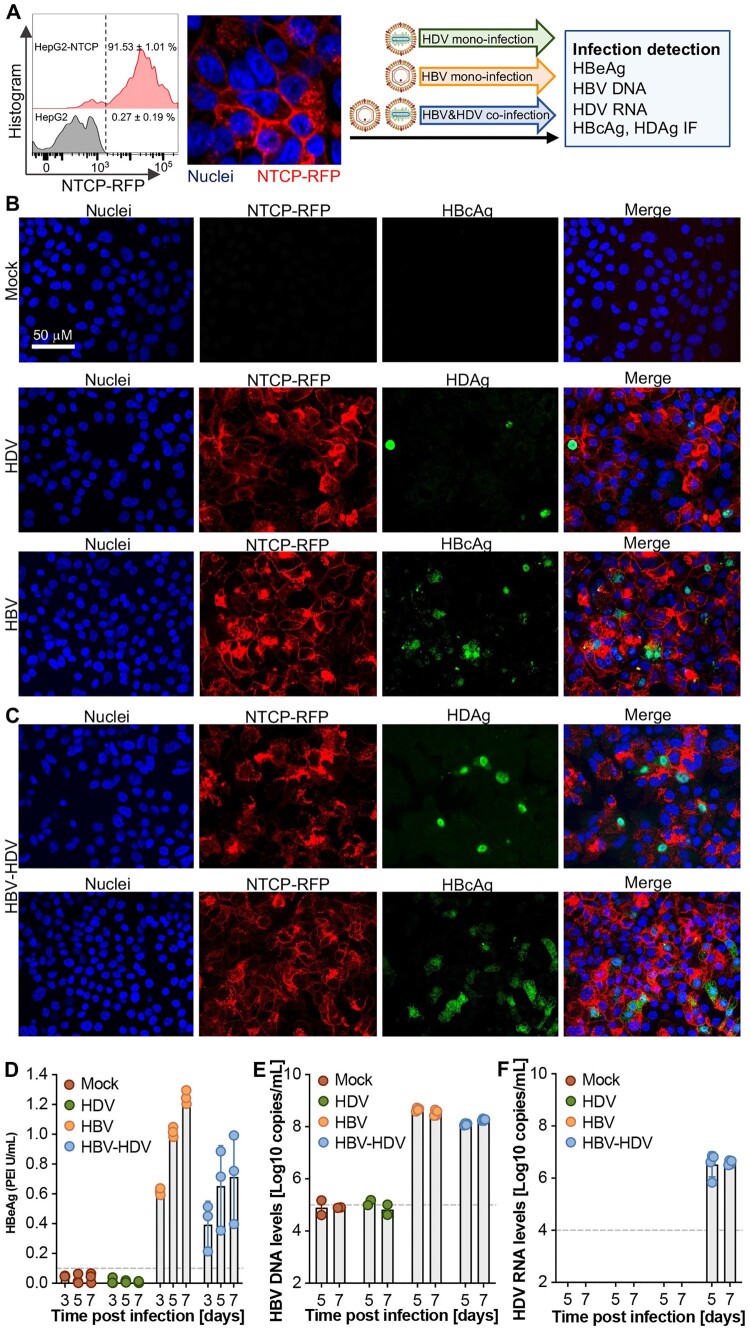
Simultaneous detection of HBV and HDV infection *in vitro*. (**A**) The HepG2-NTCP infectious cell culture model was characterized by flow cytometry and confocal imaging and the cells were used for HDV or HBV mono-infection and HBV&HDV co-infection, respectively. Representative images for immunofluorescence (IF) of HBcAg for the mock control (**B,** top), HBV single infection (**B**, middle), HDV single (**B**, down) and HBV-HDV coinfection (**C**) by detecting HBcAg and HDAg, respectively. Quantification of HBeAg levels (**D**) by ELISA and HBV DNA (**E**) and HDV RNA (**F**) copy numbers by (RT-)qPCR in the supernatants at the indicated timepoints. The data are shown as mean ± SD of 3 independent experiments.

*The HBV and HDV dual channel quantification system can be used to investigate HBV and HDV co-infection in vitro.* Next, we employed the previously established HepG2-NTCP in vitro infection system [17] to assess the functionality of our HBV and HDV dual channel quantification system. NTCP-RFP expression and membrane localization were validated through flow cytometry and confocal imaging, after which the cells were utilized for HBV mono-infection, HDV mono-infection, and HBV-HDV co-infection experiments (Figure 4(A)). To verify authentic infection, immunofluorescence assays targeting HBcAg and HDAg were conducted at 5 days post-infection, with the antibodies validated in the transfection system (Supplementary Fig.5). Consistent with previous observations [27, 28], HDV established infections in HepG2-NTCP cells (Figure 4(B), middle), while significant HBcAg-positive staining was observed in the HBV mono-infection group (Figure 4(B), bottom). In the HBV and HDV co-infection scenario, both HBcAg and HDAg were detected (Figure 4(C)).

Successful HBV infection in both the HBV mono- and HBV-HDV co-infection groups was further confirmed by the gradual increase in HBeAg levels in the supernatants (Figure 4(D)). Subsequently, HBV and HDV dual channel quantification was performed using supernatants collected at days 5 and 7 post-infection. The results revealed that the dual channel quantification system can efficiently quantify HBV DNA and HDV RNA simultaneously in the HBV-HDV co-infection group, whereas only HBV DNA was detected in the HBV mono-infection group (Figure 4(E,F)). This underscores the robust applicability of our quantification system for *in vitro* infection assays, opening the possibility for concurrent monitoring of HBV and HDV dynamics during co-infection scenarios.

**Figure 5.**
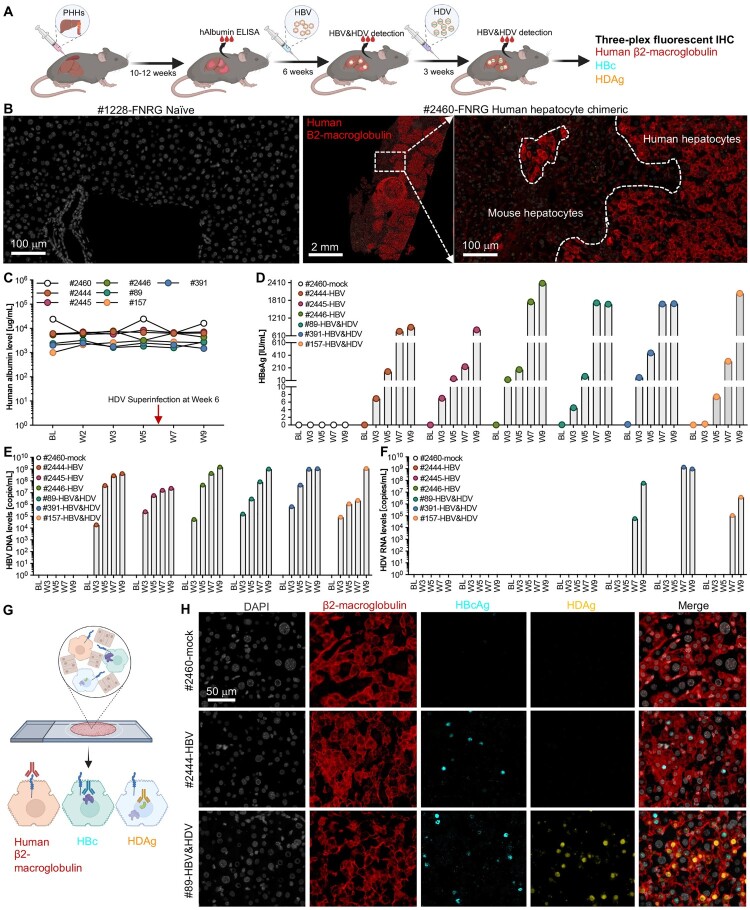
Simultaneous detection of HBV and HDV infection in the human liver chimeric mice. (**A**) Schematic of the experimental procedures of HBV-HDV superinfection in human liver chimeric mice. (**B**) Characterization of the chimeric mouse liver with human hepatocytes by human-specific β2-microglobulin fluorescent immunohistochemistry. Naïve FNRG mouse was used in parallel as a negative control. Human albumin (hAlb) (**C**), HBsAg (**D**) (both by ELISA), HBV DNA (**E**) and HDV RNA (**F**) were quantified in the sera of human liver chimeric mice at the indicated timepoints in mock, HBV mono- and HBV-HDV superinfected mice. BL, base line. (**G**) Schematic of the multiplex fluorescent IHC analysis of human β2-macroglobulin, HBc and HDAg. (**H**) Representative images of multiplex fluorescent IHC of the liver sections 9 weeks post infection. HBc-positive signals are shown as cyan, human β2-macroglobulin as red, and HDAg as yellow.

*The HBV and HDV dual channel quantification system can be used to investigate HBV and HDV infection in vivo in human liver chimeric mice.* To evaluate the performance of our HBV and HDV dual-channel quantification system *in vivo*, we generated human liver chimeric FNRG mice for HBV infection and HBV-HDV superinfection (Figure 5(A)). Liver tissue from human liver chimeric mice was histologically characterized using human-specific β2-macroglobulin *in situ* staining (Figure 5(B)) to visualize human hepatocyte engraftment. Mice with human albumin levels exceeding 1000 µg/mL at 10–12 weeks post-human hepatocyte transplantation were selected for mock infection (*n* = 1), HBV mono-infection (*n* = 3), and HBV-HDV superinfection (*n* = 3). Human albumin levels were monitored longitudinally (Figure 5(C)). Increases in serum HBsAg levels indicated successful HBV infection in both HBV mono- and HBV-HDV superinfection groups (Figure 5(D)), with detectable circulating HBV DNA levels at 3 weeks post-infection (Figure 5(E)).

At week 6 post-HBV infection, when the HBV loads exceeded 1E + 6 copies/mL in mouse serum, the mice in the HBV-HDV superinfection group were intraperitoneally inoculated with HDV (5E + 8 copies/animal). Utilizing our HBV and HDV dual-channel quantification system, only HDV RNA was detected in the HBV-HDV superinfection group (Figure 5(F)), indicating the suitability of the dual-channel quantification system for accurate HBV and HDV detection *in vivo*.

Finally, multiplex fluorescent immunohistochemistry was employed to simultaneously detect human β2-macroglobulin, HBc, and HDAg on the same tissue section simultaneously (Figure 5(G)). Human β2-macroglobulin staining confirmed robust human hepatocyte engraftment in mouse livers (Figure 5(H)). Consistent with serological viral markers, specific HBcAg staining was observed in both HBV mono- and HBV-HDV superinfection mice, while HDAg was detected exclusively in liver of the HBV-HDV superinfection groups (Figure 5(H)). This comprehensive analysis reaffirms the successful establishment of the HBV-HDV superinfection model and highlights the dual-channel quantification system's efficacy for accurate detection and monitoring of HBV and HDV dynamics in an *in vivo* setting.

**Figure 6.**
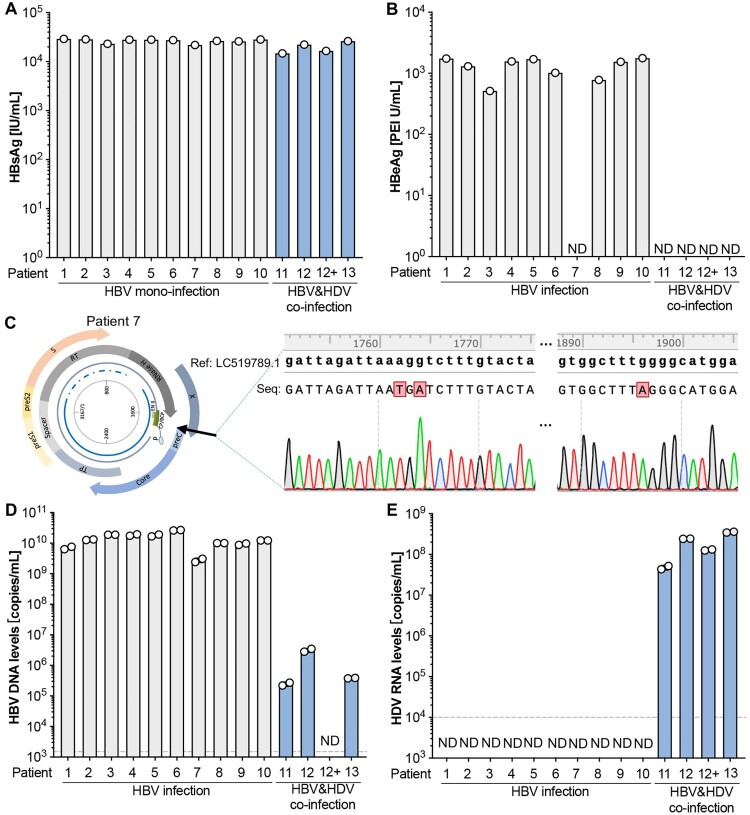
The dual fluorescence channel HBV/HDV detection system can reliably quantify HBV and HDV genome copy numbers in serum samples from HBV mono- and HBV-HDV coinfected patients. HBsAg (**A**) and HBeAg (**B**) levels in the sera from HBV+ (*n* = 10) and HBV-HDV dually infected patients (*n* = 4). (**C**) HBV genome cloning and sequencing of patient 7. A reference sequence (NCBI access number LC519789.1) was used for the comparison. Quantification of HBV DNA (**D**) and HDV RNA (**E**) copy numbers by the dual fluorescence channel HBV-HDV quantitative detection system. The dash lines show the low limit of the detection. ND, not detective.

*Preclinical assessment of the dual fluorescence channel HBV/HDV quantitative detection system on serum from patients with HBV mono-infection or HBV-HDV coinfection.* We extended the evaluation of the dual fluorescence channel HBV-HDV quantitative detection system to patient samples, including those from patients with HBV mono-infection (*n* = 10) and HBV-HDV coinfection (*n* = 3). The HBV-HDV coinfected patients were undergoing antiviral therapy with the first-line treatment drug entecavir (ETV, patient 12) or tenofovir disoproxil fumarate (TDF, patient 13) for an unspecified duration. Patient 12 had two blood draws at month 6 and month 17 post ETV therapy. HBsAg tests indicated that all patients exhibited very high levels (>10,000 IU/mL) of HBsAg, and the nucleos(t)ide analogue (NA) treatment did not significantly reduce HBsAg levels (Figure 6(A)). However, after ETV or TDF treatment, none of the HBV-HDV coinfection patients showed detectable HBeAg in the serum (Figure 6(B)), aligning with clinical observations that prolonged nucleos(t)ide analogue therapy can lead to low levels or even undetectable HBV DNA and HBeAg seroconversion [3]. Notably, patient 7 displayed undetectable HBeAg even without antiviral treatment.

To understand the molecular basis for the apparent absence of HBeAg expression in patient 7, we cloned the HBV genome from this patient. Sequencing results revealed A1762T and G1764A mutations in the core promoter (CP), basal core promoter (BCP), and Enhancer II overlapping gene expression regulatory regions and the G1896A mutation in the precure region (Figure 6(C); Supplementary Table 3). We and others have identified these regions as crucial for HBeAg production, and G1896A mutation here can abrogate HBeAg expression, while T1762A and A1764G back mutations can significantly restore HBeAg levels [19].

Using the dual fluorescence channel HBV-HDV quantitative detection system, we observed high levels of HBV DNA in all 10 patient samples without anti-HBV treatment, while patients in the HBV-HDV coinfection group undergoing NA therapy exhibited much lower (>3 log) or undetectable (patient #12) HBV DNA levels (Figure 6(D)). As expected, the dual-channel system selectively detected and quantified HDV RNA levels only in the HBV-HDV coinfection patients, not in the HBV mono-infection samples (Figure 6(E)). This underscores the system's efficacy in accurately distinguishing and quantifying HBV and HDV in clinical samples, providing valuable insights into the virological dynamics of coinfection and the impact of antiviral therapy.

## Discussion

The prevalence of HDV is likely underestimated due to the limited application of reliable HDV RNA detection in HBV patients. HDV infection is closely associated with HBV infection, indicating that HDV can only coexist in individuals who are either HBV infected or at least HBsAg positive. Given the persistent challenge of achieving HBsAg clearance for HBV patients, they remain at a high risk of HDV superinfection throughout their lives. Therefore, monitoring HBV DNA and potential HDV RNA is crucial.

Notably, the co-infection of HBV and HDV can expedite the progression from chronic hepatitis to end-stage liver diseases, significantly increasing morbidity and mortality [11]. Early diagnosis of HDV infection is paramount, and universal screening in HBV patients is both feasible and cost-effective. The HBV-HDV dual quantification system we developed offers a timely and cost-efficient solution. Through a single quantification process, we can obtain two readout reports, allowing simultaneous monitoring of HBV and HDV levels.

Presently, HDV diagnosis heavily relies on blood tests for HDV antibodies (IgG and IgM), though this method fails to distinguish ongoing from past infections. Moreover, serological detections often lack sensitivity and quantification capabilities [29]. The gold standard for diagnosing ongoing HDV infection is the presence of HDV RNA and viremia. Several RT-qPCR-based in-house HDV quantification methods have been developed [30–33], and more recently, a CRISPR/Cas13a-based HDV RNA detection technology with high sensitivity has been introduced [34].

However, existing assays typically focus on a single target, quantifying HDV RNA alone, thereby limiting the ability to monitor dynamic interactions between HBV and HDV. Furthermore, some assays employ HDV DNA plasmids or cDNA as standards for HDV RNA quantification, raising concerns about the calibration of reverse transcription efficiency. This inconsistency makes the reliability of HDV RNA quantification from different labs questionable and incomparable.

In this study, we addressed these issues by generating a full-length genomic (minus strand) HDV RNA standard through *in vitro* transcription. This standard closely mimics HDV genome RNA purified from virions, serving as a robust calibration tool for reverse transcription and amplification processes during quantification. It is important to note that while we established theoretically recognized calibration standards for HDV RNA quantification, we did not calibrate them with the international standard from the World Health Organization (WHO) [35]. Further calibration may be necessary in the future to obtain an international unit (IU) readout for this dual detection system.

In the context of HBV DNA quantification within our dual detection system, we have previously developed specific over-gap amplification primers designed for HBV rcDNA. These primers selectively target infectious HBV with rcDNA genomes, excluding non-infectious HBV particles that only possess short minus-strand DNA [2, 36]. This design proves particularly valuable for accurately quantifying the viral load of HBV, especially in samples from patients undergoing Nucleos(t)ide Analog (NAs) therapy [2].

Moreover, our HBV detection system effectively eliminates the detection of HBV RNA. Prior research has shown that encapsidated and secreted HBV RNA are 3’ terminus truncated, rendering our HBV detection system capable of valid amplification and subsequent quantification exclusively for HBV rcDNA [24, 25]. Consequently, the integration of this HBV rcDNA-specific detection into the HBV-HDV dual detection system enhances the specificity of our HBV DNA quantification in this one-step reverse transcription and quantification amplification combined system.

The HBV-HDV dual system is designed to be compatible for pan-genotypic detection of both HBV and HDV. HBV DNA polymerase (pol) lacks proofreading function (3′ exonuclease activity), resulting in remarkable genetic heterogeneity, with at least nine classified genotypes (A to I) [19]. HDV, with its high genome variety, exhibits sequence heterogeneity of up to 30% among different genotypes, which are differently distributed across the globe [23]. A pan-genotypic and universal detection method is advantageous for targeting and identifying different genotypes worldwide.

Through phylogenetic analysis and sequence alignment, we identified highly conserved regions for HBV and HDV, allowing us to design primers and probes, respectively. Leveraging the affinity plus probe technology, we enhanced structural stability, increasing the hybridization melt temperature, even with a shorter sequence in limited conserved regions. Notably, in the HDV forward primer, a C/T polymorphism between genotype 4 and other genotypes necessitated the use of a degenerate base Y to ensure the pan-genotypic detection feature for HDV.

The HBV-HDV dual detection system has numerous applications, providing critical insights into interactions between these two viruses. The readouts of HBV DNA and HDV RNA serve as valuable markers for monitoring responses to antiviral therapies, particularly in the current era of anti-HBV spanning approximately two decades. With the approval of bulevirtide heralding a new era of anti-HDV therapy [14], the dual detection system holds promise in assessing treatment outcomes.

Using the HBV-HDV interaction through this system, we have identified its utility in *in vitro* HBV-HDV transfection and infection models. Our results indicate that HDV may impede HBV DNA replication and the expression of secreted proteins, consequently increasing intracellular core protein accumulation. Conversely, as expected, HBV appears to facilitate the release of progeny HDV virions while decreasing intracellular HDV antigens.

Upon applying the HBV-HDV dual detection system to *in vivo* samples, we consistently detected stable levels of HBV DNA and HDV RNA in plasma or serum samples from both human liver chimeric mice and patients. Notably, despite effective NA treatment suppressing HBV DNA to low or undetectable levels, HDV RNA levels remained maintained at high levels. In the future it will be import to validate this detection system in larger patient populations known or suspected to be infected with HBV and HDV. Looking ahead, leveraging our HBV-HDV dual detection system, and incorporating automated nucleic acid extraction into the protocol, holds the potential to establish a standard, commercial, and universal assay for simultaneous quantification of HBV DNA and HDV RNA.

## Supplementary Material

HBV_HDV_detection_Supplementary_data_YLAP20240317

## Data Availability

Raw data for all figures shown in this manuscript can be found at Princeton DataSpace 10.34770/s4zx-ab39.
