# Supplementary material for: Development of a dual channel detection system for pan-genotypic simultaneous quantification of hepatitis B and delta viruses
Source: Emerg Microbes Infect. 2024 Apr 30;13(1):2350167. doi: 10.1080/22221751.2024.2350167 (PMC11095294; doi:10.1080/22221751.2024.2350167)
Supplement: HBV_HDV_detection_Supplementary_data_YLAP20240317 [file TEMI_A_2350167_SM9169.docx]

Supplementary Information

for

**Development of a dual channel detection system for pan-genotypic simultaneous quantification of hepatitis B and delta viruses**

Yongzhen Liu^1^, Stephanie Maya^1^, Sebastian Carver^1^, Aoife K. O’Connell^2^, Anna Zen^2^, Hans P. Gertje^2^, Kathleen Seneca^3^, Ronald G. Nahass^3^, Nicholas Crossland^2,4,5^, Alexander Ploss^1, *^

^1^ Department of Molecular Biology, Princeton University, Princeton, NJ 08540, USA

^2^ National Emerging Infectious Diseases Laboratories, Boston University, Boston, MA, USA

^3^ Infectious Disease Care, 105 Raider Boulevard, Hillsborough, NJ 08844, USA.

^4^ Department of Pathology and Laboratory Medicine, Boston University Chobanian & Avedisian School of Medicine, Boston, MA, USA

^5^ Department of Virology, Immunology, & Microbiology, Boston University Chobanian & Avedisian School of Medicine, Boston, MA, USA

* Correspondence should be addressed to A.P. ([aploss@princeton.edu](mailto:aploss@princeton.edu))

**Keywords:** Hepatitis B virus; hepatitis delta virus; viral hepatitis; viral detection; humanized mice

**Supplementary Table 1. Primer and probe sequences used in this study**

| Primer/Probe | 5’ to 3’ |
| --- | --- |
| HBV-qPCR-F | GGAGGCTGTAGGCATAAATTGG |
| HBV-qPCR-R | CACAGCTTGGAGGCTTGAAC |
| HBV-probe | FAM-AGATG+ATTA+GGCAGA+GGTGAAAAAG-IB^®^FQ |
| HDV-qPCR-F | GCGCCGGCYGGGCAAC |
| HDV-qPCR-R | TCCTCTTCGGGTCGGCATG |
| HDV-probe | TAMRA-GGTCC+G+ACCTGGGC+ATCCG-IB^®^RQSp |

**Supplementary Table 2**. Antibodies and antigen retrieval conditions for the fluorescent assay performed in this study.

| **Seq.** | **Antigen Target** | **Species Origin** | **Clone** | **Manu-**  **facturer** | **Cata-log** | **Prim. ab dilution** | **Fluorophore** | **Incubation Period** | **Incubation Temperature** | **Opal Dilution** | **Notes** |
| --- | --- | --- | --- | --- | --- | --- | --- | --- | --- | --- | --- |
| Three-plex |  |  |  |  |  |  |  |  |  |  |  |
| 1 | HBcAg | Rabbit | N/A | LSbio | C312204 | 1/100 | Opal 480 | 2 hours | Room temperature | 1/30 | Used with Discovery Amplification HQ Kit, 12 minute incubation |
| 2 | HDAg* | Rabbit | N/A | N/A | N/A | 1/700 | Opal 570 | 1 hour | Room temperature | 1/150 |  |
| 3 | β2-microglobulin | Rabbit | D8P1H | Cell signaling | 12851S | 1/600 | Opal 690 | 1 hour | Room temperature | 1/90 |  |

*The anti-HDAg antibody was a gift from Dr. John Taylor, Fox Chase Cancer Center, Philadelphia, PA.

**Supplementary Table 3**. Summarized mutations of HBV genome isolated from patient 7 spanning nt 1140 - nt 2452 region.

| **Nucleotide position*** | **HBV gene region** | **Mutation** | **Amino acid change** |
| --- | --- | --- | --- |
| 1762 | Enhancer II/core promoter/basal core promoter/HBx | A → T | HBx codon 130  K → M |
| 1764 | Enhancer II/core promoter/basal core promoter/HBx | G → A | HBx codon 131  V → I |
| 1835 | core promoter/basal core promoter/HBx/precore | C → T | HBx codon 154  Samesense mutation  precore codon 8  Samesense mutation |
| 1896 | precore | G → A | precore codon 28  W → **stop codon** |
| 2044 | precore/core | C → T | precore codon 77  Samesense mutation  core codon 48  Samesense mutation |
| 2092 | precore/core | G → T | precore codon 93  E → D  core codon 64  E → D |
| 2093 | precore/core | T → C | precore codon 94  Samesense mutation  core codon 64  Samesense mutation |
| 2224 | precore/core | T → C | precore codon 137  Samesense mutation  core codon 108  Samesense mutation |
| 2363 | precore/core | T → A | precore codon 184  S → T  core codon 155  S → T |

*The nucleotide position was alignment to a wide-type genotype C genome (NCBI access # LC519789.1)

**Supplementary Figures**

**Supplementary Figure 1**. (**A**) HDV 1.0x genome was amplified from the psvL(D3) plasmid and cloned into a TOPO vector. The HDV 1.0x genome fragment with T7 promoter was then amplified, purified, and used for *in vitro* RNA transcription (IVT). The resultant HDV RNA was then treated with DNase I to degrade the DNA template and the purity was confirmed by gel electrophoresis. (**B**) Generation of HBV minicircle DNA. (**C**) Supercoiled HBV minicircle DNA or its EcoR1 linearized version was confirmed by DNA gel electrophoresis. (**D**) The generated HBV circle DNA was characterized by transfecting to HepG2 cells and HBV DNA and HBsAg quantified in the supernatants by qPCR and ELISA, respectively.

**Supplementary Figure 2**. HBV DNA (**A**) and HDV RNA (**B**) amplification plots show the fluorescence signal amplification (shown as the ΔRn) with the amplification cycles and the corresponding standard curves.

**Supplementary Fig. 3.** (**A**) Phylogenetic analysis of HBV genomes from different genotypes. Three representative genomes for each genotype were utilized. The table shows the % identity (right, colored by light blue) and distance (left, colored by red) between any two genomes. The darker the color is, the higher identity or distance. (**B**) Phylogenetic analysis of HDV genome of different genotypes. Three representative genomes for each genotype were included in the analysis except for gts 1 and 6 due to lack of reliable full-length sequences. The table shows the % identity (right, colored by light blue) and distance (left, colored by red) between any two genomes. The darker the color is, the higher identity or distance. The NCBI accession numbers are shown in the figure.


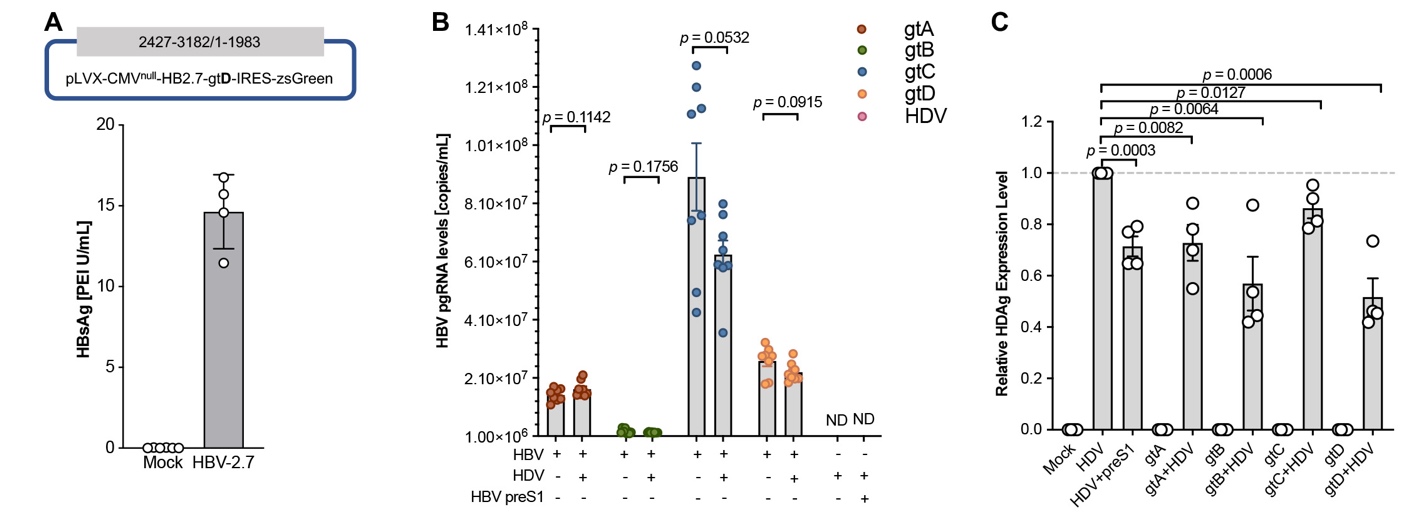


**Supplementary Figure 4**. (**A**) HBV preS1 (genotype D, ayw) region was amplified and was cloned into a pLVX expression vector. The expression of HBV surface protein was validated by ELISA. (**B**) HBV pgRNA detection in the supernatants after HBV single transfection, HBV-HDV co-transfection and HDV-HBV preS1 co-transfection. ND, not detected. (**C**) Quantification of HDV antigen level by western blot after HBV single transfection, HBV-HDV co-transfection and HDV-HBV preS1 co-transfection. The band intensity was valued by Image J. Statistical significance was determined applying a two tailed student t test.


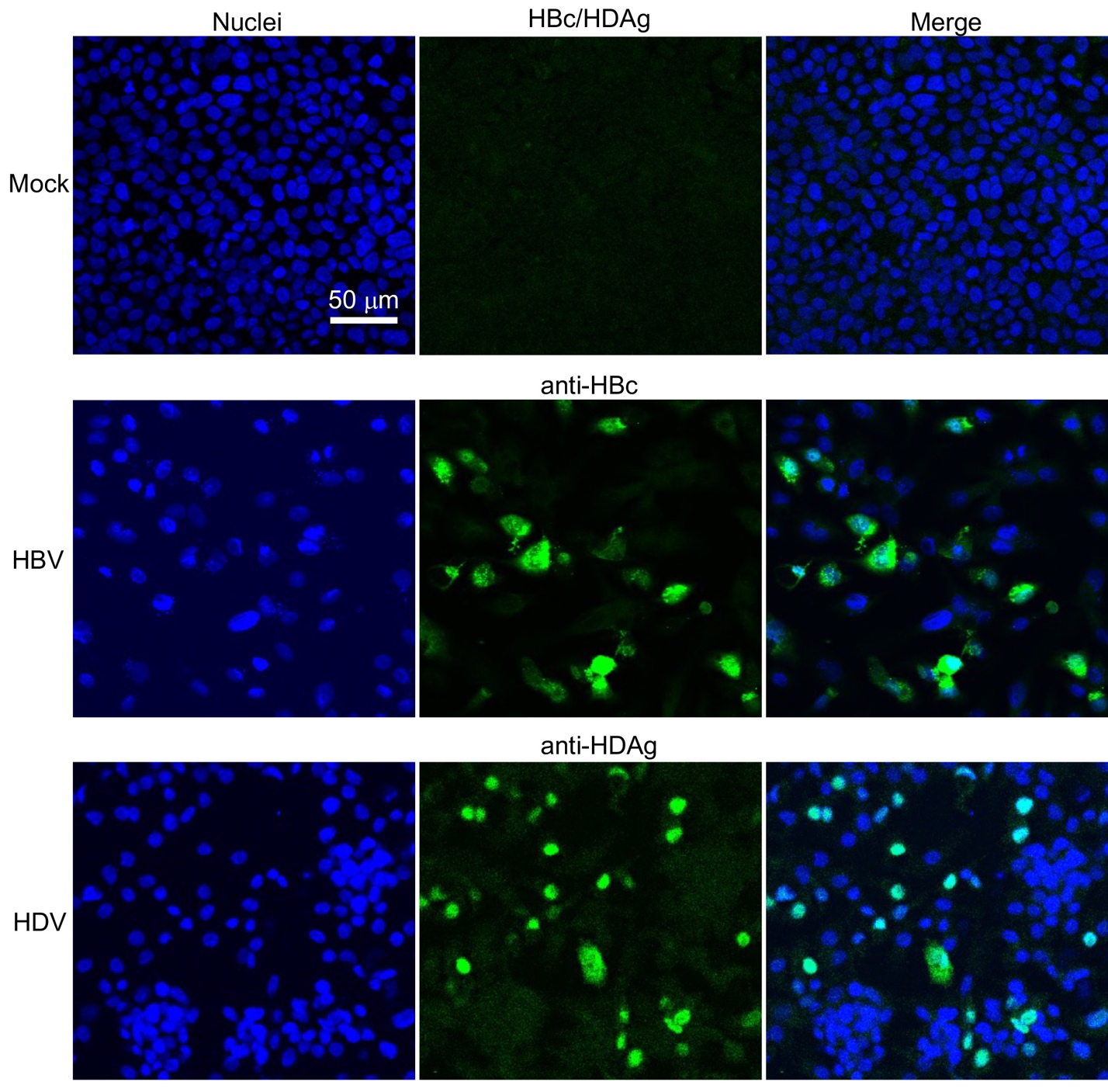


**Supplementary Figure 5**. Representative immunofluorescence (IF) images of HBc and HDAg after mock transfection, or transfection with plasmids encoding an HBV 1.3x infectious clone (genotype D), or 3x version of a HDV genome (psvL(D3)) into HepG2 cells. Images from different fluorescence channels (405 nm and 488 nm) and merged images are shown.
